# Rare Ocular Involvement in a Newly Diagnosed AIDS Patient With Diffuse Kaposi’s Sarcoma

**DOI:** 10.7759/cureus.8502

**Published:** 2020-06-08

**Authors:** Luis C Segura, Anuj Goel

**Affiliations:** 1 Internal Medicine, Methodist Health System, Dallas, USA

**Keywords:** kaposi's sarcoma, aids, hiv, ophthalmology, humans herpes virus 8, immune reconstitution syndrome, haart, iris, infectious disease, bleomycin

## Abstract

Kaposi’s sarcoma (KS) is a cancer that often affects individuals with human immunodeficiency virus, acquired immunodeficiency syndrome (AIDS), those who have received an organ transplant, or others who are immunocompromised. KS is a vascular tumor that most often presents in cutaneous sites, including the lower extremities, oral mucosa, and genitalia. Literature regarding KS with ocular involvement is scarce. We present a rare case in which a patient diagnosed with AIDS-associated KS exhibited ocular and diffuse manifestations. The lesions of cutaneous KS are frequently mistaken for an alternative diagnoses; therefore, the clinician should have a high index of suspicion for this vascular tumor in AIDS patients.

## Introduction

Kaposi’s sarcoma (KS), a vascular tumor associated with human herpes virus 8 (HHV-8) infection, is the most commonly seen neoplasm in patients with acquired immunodeficiency syndrome (AIDS) [1]. It typically presents in the lower extremities, oral mucosa, and genitalia. Early KS lesions are typically mistaken for other dermatologic conditions [2]. It is important to identify KS with biopsy and molecular testing for HHV-8 as early as possible to reduce the morbidity and mortality associated with these tumors. Ocular involvement of these vascular tumors is rarely documented [3]. We present a rare case of AIDS-associated KS presenting with ocular and diffuse manifestations. 

## Case presentation

A 23-year-old, bisexual, cisgender male presented to the emergency department (ED) with left groin swelling and perirectal pain that had been ongoing for five days. He was predominantly involved with men, and last had anal-receptive intercourse three months prior. His last human immunodeficiency virus (HIV) screen had been negative five years prior. However, on this admission he was diagnosed HIV positive (viral load of 15,171 copies/ml) and with AIDS (CD4 cell count of 8 cells/mm^3^). Abdominal imaging on presentation revealed diffuse retroperitoneal, iliac, pelvic, and inguinal lymphadenopathy and findings suggestive of proctitis. He was treated with antibiotics for the presumed proctitis; he responded well. The infectious diseases service was consulted, and the patient agreed to follow-up as an outpatient. The colorectal surgery department was also consulted for further evaluation, monitoring, and treatment. One month later, the patient was started on antiretroviral therapy (ART). At the surgeon’s office, perianal lesions were noted, which were thought to be condyloma acuminata. The decision was made to allow time for the patient’s CD4 cell count to increase prior to excision and fulguration of the presumed condylomas. 

**Figure 1:**
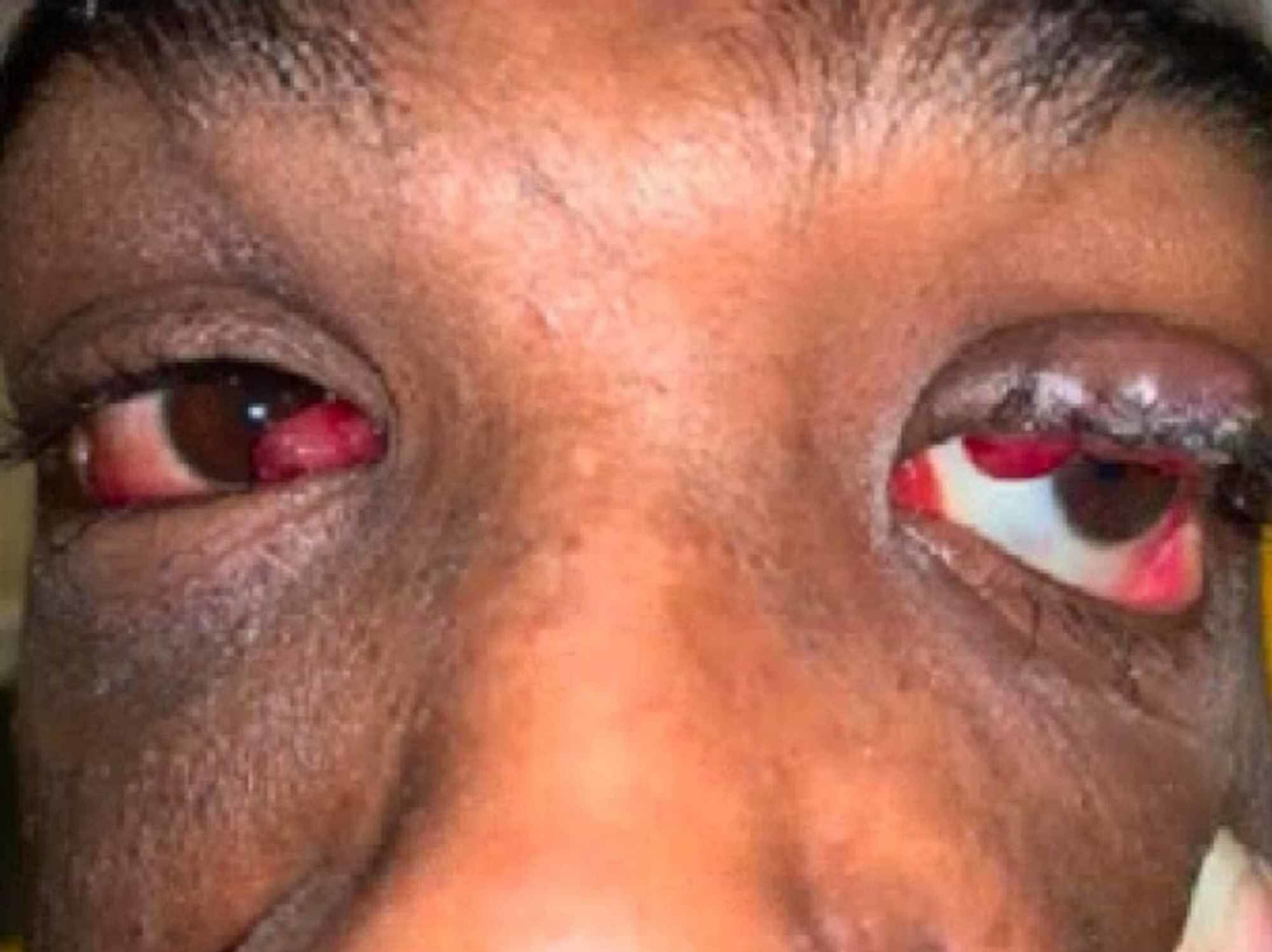
Ocular manifestations of Kaposi’s sarcoma. The patient exhibited red, nodular conjunctival masses.

**Figure 2:**
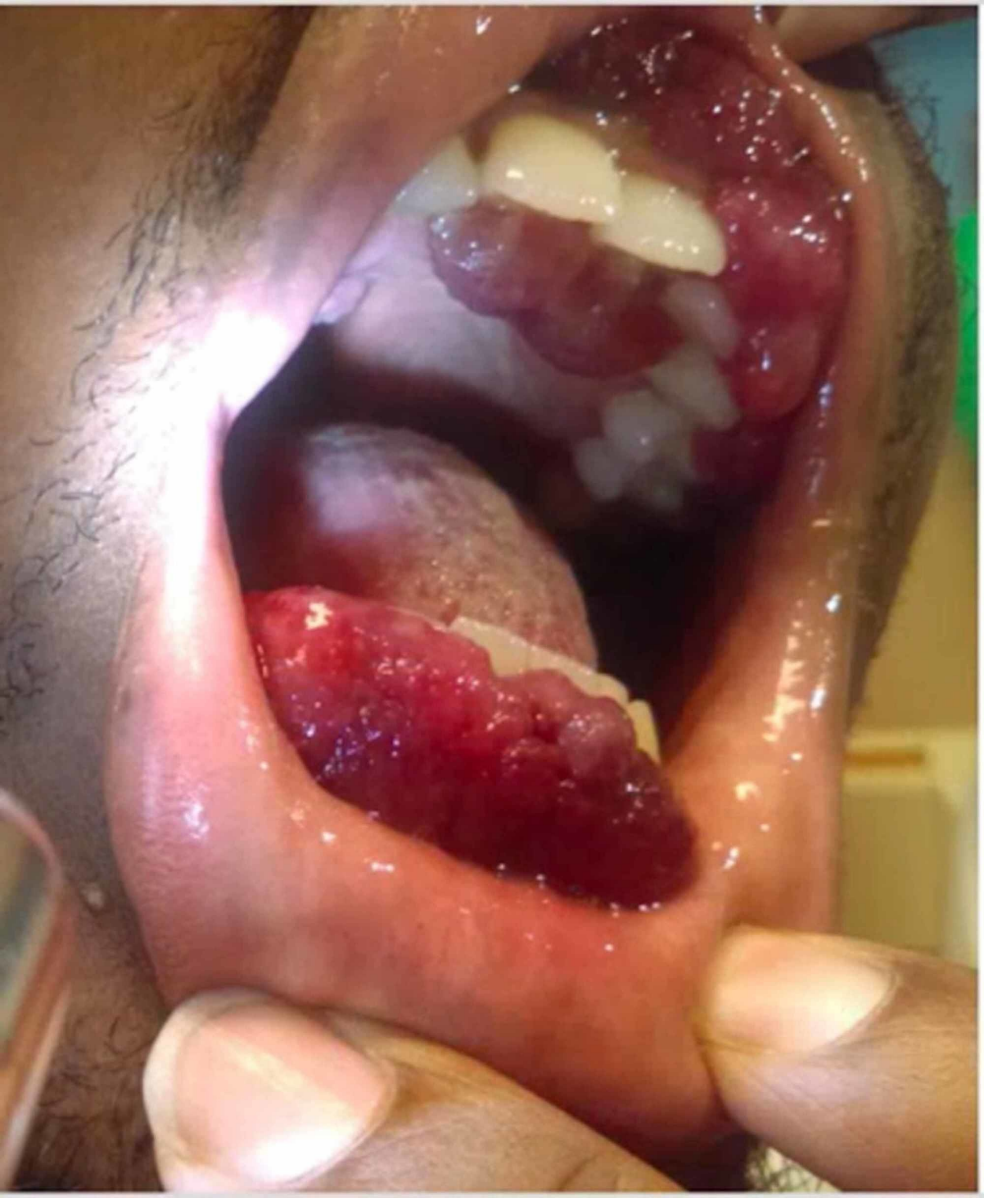
Gingival and palatal involvement of Kaposi’s sarcoma. Extensive violaceous, nodular tutors were observed on multiple gingival and palatal surfaces.

Three weeks later, the patient again presented to the ED, with diffuse myalgias and signs of sepsis. Five days prior to this second admission, he experienced conjunctival swelling (Figure 1), new gingival enlargement (Figure 2), and erythema. He was treated with broad-spectrum antibiotics.

**Figure 3:**
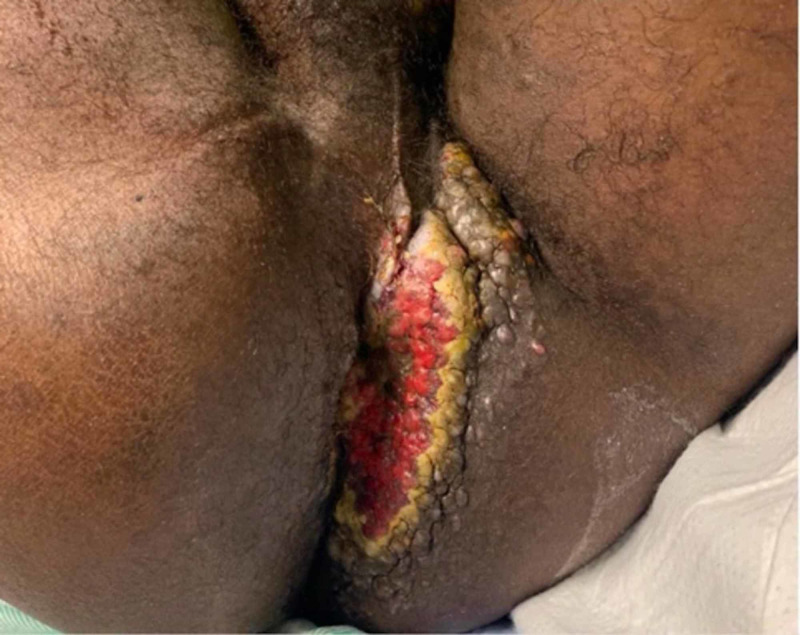
Perianal Kaposi’s sarcoma. A large, red-violaceous, irregular contour perianal mass was observed.

A CT scan of the chest demonstrated mediastinal and hilar adenopathy as well as new widespread pulmonary nodules throughout both lungs; the largest nodule measured up to 1.2 cm. He had no respiratory issues and no obvious source of infection other than presumed gingivitis and perianal cellulitis, as he had worsening of his perianal lesions (Figure 3). 

He was discharged in stable condition three days later with orders to follow up with infectious diseases and colorectal surgery. Ten days later, he experienced worsening gingival enlargement, bloody drainage from his eyes, bleeding from his perianal lesions, and new submandibular lymphadenopathy. He was sent to the ED by the infectious diseases physician who was concerned that these findings were due to KS, with cutaneous, pulmonary, and ocular involvement associated with immune reconstitution inflammatory syndrome (IRIS). 

**Figure 4:**
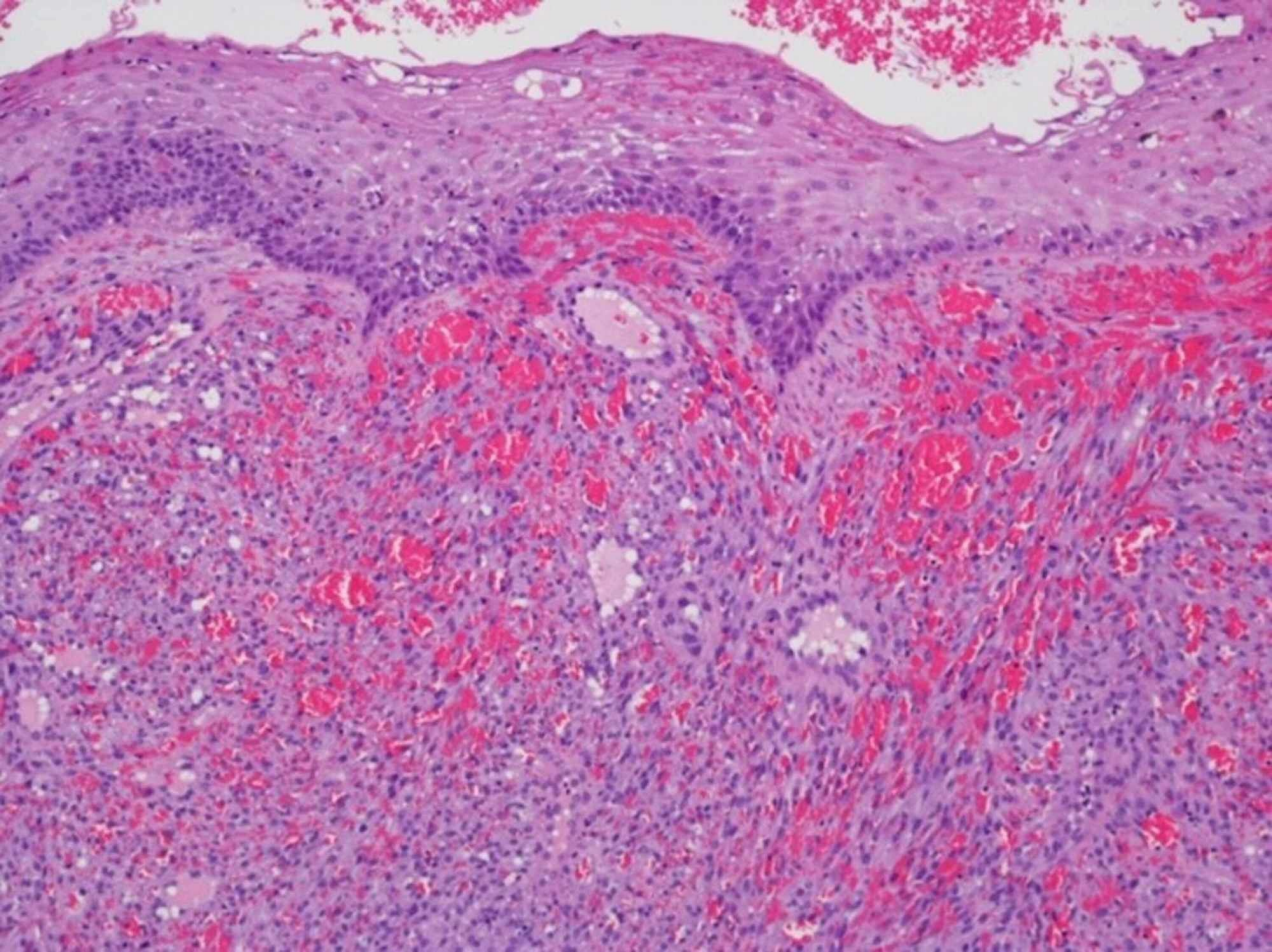
Anterior gingival biopsy of an ovoid nodule lined on the surface by squamous epithelium. A Kaposi's sarcoma tumor takes up the rest of the tissue. The tumor consists of spindled cells forming slit-like spaces. There are numerous extravasated red blood cells. The individual cells show mitotic activity, have poor defined cell borders, and ovoid, small to medium-sized nuclei with occasional pinpoint nuclei (hematoxylin and eosin stain, X100).

During this admission, multiple biopsies were obtained, including from the anterior maxillary gingiva, left axillary lymph node, and perianal lesions. Gingival biopsies showed mitotic activity with spindled cells forming slit-like spaces with numerous extravasated red blood cells (Figure 4).

**Figure 5:**
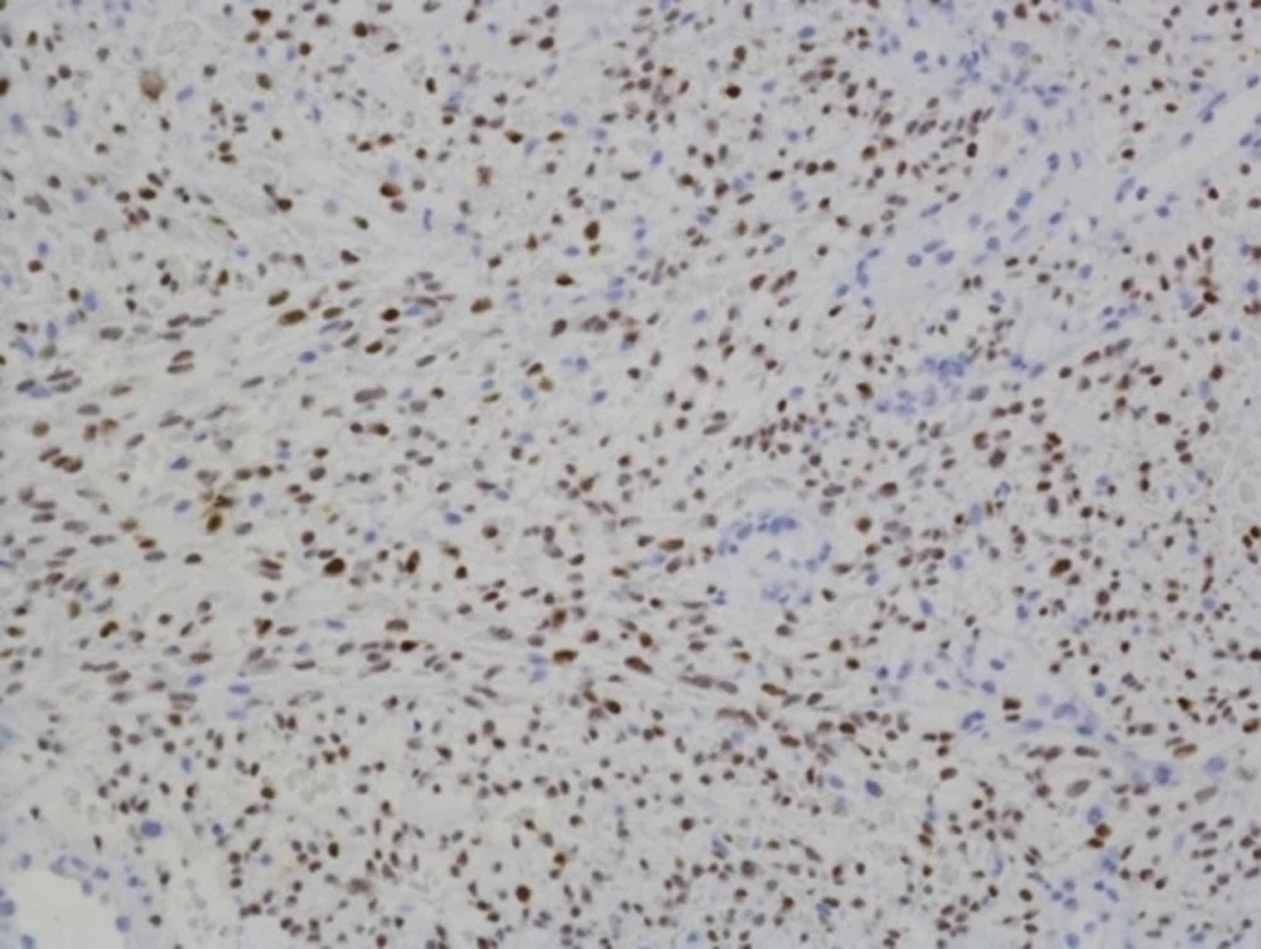
Immunohistochemistry. Gingival biopsy demonstrating immunoreactivity for HHV-8 in the tumor cell nuclei (X40). HHV-8, human herpes virus 8.

Immunohistochemical stains were strongly and diffusely positive for HHV-8 (Figure 5) and CD31 (data not shown). The left axillary node biopsy was also positive for overall tumor morphology and immunohistochemical staining patterns consistent with KS. Anal biopsy showed vascular proliferation with immunohistochemical stains demonstrating vast expression of CD31 and HHV-8. 

An ophthalmology consult was obtained for suspicion of ocular KS. Comprehensive examination showed no abnormalities of visual fields, acuity, or extraocular movements. Ocular biopsy was not obtained due to risk of bleeding. His CD4 cell count at that time was 40 cells/mm^3^ with an undetectable viral load. A hematology-oncology consultation was obtained, and the recommendation was made to start the patient on doxorubicin and continue ART. Close follow-up with infectious disease, hematology-oncology, ophthalmology, and colorectal surgery was also recommended. Because our patient did not present with upper respiratory signs, we did not pursue pulmonary node biopsies due to risk of hemorrhaging with these highly vascular tumors. Instead, we opted for surveillance with imaging and treatment.

## Discussion

KS is the most common neoplasm in patients with HIV and AIDS [1]. KS has a highly variable progression; patients may present with minimal disease to dramatic spread [2]. KS was first described in 1872 by the pathologist Moritz Kaposi and is the most common tumor arising in persons infected with HIV [2,3]. Extracutaneous extension is common, involving the oral cavity, gastrointestinal tract, lungs, and lymph nodes most frequently. 

We found few documented cases and studies of KS with ocular involvement. A study of HIV/AIDS patients reported ocular KS in 0.25% of patients with KS diagnosed at other sites [3]. Local treatment with intralesional vinblastine or bleomycin, radiotherapy, and electrochemotherapy is standard. Systemic therapy includes highly active ART (HAART) along with systemic chemotherapy such as daunorubicin and pegylated liposomal doxorubicin [4]. 

Our patient presented with anal KS lesions initially thought to be condyloma acuminata, illustrating that KS is frequently mistaken for other dermatologic conditions [5]. Through his social history, we were able to identify risk factors for HIV and diagnose him with AIDS upon presentation. Several opportunistic infections may result in cutaneous lesions that mimic KS lesions, including bacillary angiomatosis, cryptococcus, and blastomycosis. For this reason, biopsy and consultation with an infectious disease specialist is recommended by current practice guidelines [6]. Due to the diffuse presentation of KS in the current case, we felt that diagnostic workup and biopsy were important prior to discharge. 

Our patient was started on ART, with a resulting increase in his CD4 cell count; however, he developed a rapid, aggressive, and diffuse form of KS associated with IRIS that included multiple systems throughout his body. In some patients, initial development of a tumor or tumor exacerbation occurs after the initiation of ART; this may be due to IRIS [6]. Glucocorticoids, a common treatment for other forms of IRIS and/or AIDS-related pulmonary infections, can also exacerbate the tumor and should be avoided when possible [6]. No current standard therapy protocols are available, although several therapeutic options exist. Treatment decisions are based upon the extent of the disease, immune status, and concurrent complications [7]. Treatment may include local therapy, systemic HAART therapy, and/or systemic chemotherapy [7]. Due to the advanced presentation with multiorgan involvement observed in the current case, we expect our patient will be treated with the more generalized therapies, such as HAART and systemic chemotherapies. 

The outcomes for patients with KS are difficult to describe and predict due to its highly variable course, and although no randomized control trials exist, observational studies suggest that earlier detection of KS may be more beneficial [7]. We were able to obtain biopsies and multidiscipline follow-ups for our patient, who remained stable throughout his hospitalization and at discharge. We anticipate that the patient will receive appropriate follow-up care and treatment with ART, chemotherapy, and surveillance imaging.

## Conclusions

In patients with HIV and/or AIDS, a high index of suspicion for KS should be maintained for masses, bleeding tissue, oral mucosa changes, and/or gingival enlargement. There can also be involvement in atypical sites, such as the conjunctiva, as in our patient. Although often misdiagnosed, KS can be confirmed through biopsy and molecular testing for HHV-8.
